# Luciferase shRNA Presents off-Target Effects on Voltage-Gated Ion Channels in Mouse Hippocampal Pyramidal Neurons

**DOI:** 10.1523/ENEURO.0186-17.2017

**Published:** 2017-10-11

**Authors:** Yuto Hasegawa, Wenjie Mao, Sucharita Saha, Georgia Gunner, Jenya Kolpakova, Gilles E. Martin, Kensuke Futai

**Affiliations:** Brudnick Neuropsychiatric Research Institute and Program in Neuroscience, University of Massachusetts Medical School, Worcester, MA 01604

**Keywords:** ion channels, luciferase, off-target effects, RNA interference, short hairpin

## Abstract

RNA interference (RNAi) is a straightforward approach to study gene function from the *in vitro* cellular level to *in vivo* animal behavior. Although RNAi-mediated gene knockdown has become essentially routine in neuroscience over the past ten years, off-target effects of short hairpin RNAs (shRNAs) should be considered as the proper choice of control shRNA is critical in order to perform meaningful experiments. Luciferase shRNA (shLuc), targeting firefly luciferase, and scrambled shRNAs (shScrs) have been widely used as controls for vertebrate cell research. However, thorough validation of control shRNAs has not been made to date. Here, we performed thorough physiological and morphological studies against control shRNAs in mouse hippocampal CA1 pyramidal neurons. As expected, all control shRNAs exhibited normal basal synaptic transmission and dendritic morphology. However, to our surprise, shLuc exerted severe off-target effects on voltage-gated ion channel function, while the shScr had no detectable changes. These results indicate that thorough validation of shRNA is imperative and, in the absence of such validation, that shScr is the best available negative control for gene knockdown studies.

## Significance Statement

RNA interference (RNAi), a process through which small RNAs induce sequence-specific post-transcriptional gene silencing, is widely recognized as one of the most ideal tools not only for functional genomics but also for therapeutic applications. Ensuring the specificity of small interfering RNAs (siRNAs) for specific messenger RNAs is critical, as off-target effects of siRNA can compromise the interpretation of data. Since the existence of off-target effects has been suggested in the past, it is critical to unequivocally establish that siRNAs do not present unintended effects on the biophysical properties of neurons. Here, we found that luciferase short hairpin RNA (shRNA), but not scrambled shRNA (shScr), exhibited off-target effects on voltage-gated ion channels, indicating that careful evaluation is required for studies using luciferase shRNA (shLuc) and siRNA in general.

## Introduction

Since the finding of RNA interference (RNAi; Fire et al., 1998), RNAi-mediated gene knockdown has become one of the most straightforward and high throughput approaches to address gene functions from the cellular level to the animal level. Although this approach is technically straightforward, off-target effects have been repeatedly reported and represent a major concern when using RNAi gene knockdown technology. Three off-target mechanisms have been described: (1) microRNA-like regulation through sequence complementary to the small interfering RNA (siRNA) seed region (Jackson et al., 2003; Birmingham et al., 2006; Jackson et al., 2006a,b); (2) Toll-like receptor-mediated immune stimulation (Sledz et al., 2003; Kariko et al., 2004); (3) oversaturation of endogenous RNAi machinery by siRNA and short hairpin RNA (shRNA) transfection (Grimm et al., 2006; Khan et al., 2009). Therefore, it is important to perform multiple layers of confirmatory experiments to validate the effect(s) of RNAi, such as verifying phenotypes by independent multiple siRNAs, rescuing phenotypes by exogenous transgene expression and including nonspecific RNAi control(s) (Cullen, 2006).

shRNAs, the most widely used double-stranded RNAs, are processed to siRNAs by Dicer and silence target genes along the RISC-mediated RNAi pathway. shRNA directed against firefly luciferase (shLuc) has been widely used as a control in mammalian cells. To date, >70 publications used shLuc as a control. However, control shRNAs, including shLuc, have not been fully validated. Alvarez et al. (2006) reported the off-target effects of shLuc in hippocampal CA1 pyramidal neurons. Their findings indicate that transfection of shLuc caused dysregulation of spine density and dendritic complexity, and concomitant reduction of excitatory and inhibitory synaptic transmission. Despite these striking results, shLuc continues to be used as a control (Hoogenraad et al., 2010; Wakita et al., 2011; Chen et al., 2014a,b), indicating the need for a thorough validation of control shRNAs in cellular function.

Here, we have performed a detailed characterization of two control shRNAs on neuronal function and morphology, and demonstrate that shLuc has considerable off-target effects on voltage-gated ion channels without exhibiting any synaptic or morphologic defects. In contrast, nonsilencing scrambled shRNA (shScr) exhibited no abnormal neuronal functions and morphology. This study highlights the importance of thoroughly validating shRNAs and proposes shScr as a negative control appropriate for gene knockdown studies in mammalian cells.

## Materials and Methods

### Animals

All animal protocols were approved by the Institutional Animal Care and Use Committee of the University of Massachusetts Medical School. Male and female C57BL6 mice were used.

### DNA constructs

The human H1 promoter-based pSuper Luciferase-RNAi construct has been previously described (Zhang and Macara, 2006) and targets the sequence 5´-CGTACGCGGAATACTTCGA-3´. pGIPZ-shScr (Dharmacon, #RHS4346) targets the sequence 5´-ATCTCGCTTGGGCGAGAGTAAG-3´. Dharmacon nontargeting scrambled sequence was designed using a proprietary algorithm to ensure the sequence will not target any annotated gene in human, mouse, or rat. EGFP (Clontech) gene was sub-cloned to pCAG vector.

### Organotypic slice culture preparation and biolistic gene transfection

Mice hippocampal organotypic slice cultures were prepared from postnatal day 5–7 C57BL6 mice (both genders; Stoppini et al., 1991; Futai et al., 2007; Futai et al., 2013). Briefly, hippocampal slices (350-μm thickness) were prepared using a tissue chopper (Ted Pella, INC), and slices were cultured in a CO_2_ incubator at 35°C. Neurons were transfected at days *in vitro* 4–6 using a biolistic gene gun (Bio-Rad; Lo et al., 1994) with 1.6-μm gold particles (10 mg per ∼50 bullets) coated with cDNAs: shRNA vector, pCAG and pCAG-EGFP (45:45:10 μg), and were assayed 5 d after transfection, unless otherwise noted.

### Electrophysiology

The recording chamber was filled with extracellular solution containing 119 mM NaCl, 2.5 mM KCl, 4 mM CaCl_2_, 4 mM MgCl_2_, 26 mM NaHCO_3_, 1 mM NaH_2_PO_4_, and 11 mM glucose, gassed with 5% CO_2_/95% O_2_, pH 7.4. For whole-cell recordings, thick-walled borosilicate glass pipettes (Warner Instruments) were pulled to a resistance of 2–4 MΩ. Na^+^ currents were measured in the presence of K^+^ and calcium (Ca^2+^) channel blockers, tetraethylammonium (TEA; 30 mM, Sigma), 4-amynopyridine (4-AP; 0.5 mM, Sigma), and CdCl_2_ (100 mM) in extracellular solution. To measure K^+^ currents, tetrodotoxin (TTX; 1 μM) and CdCl_2_ were applied to extracellular solution to block Na^+^ and Ca^2+^ channels.

Current-clamp recordings were performed with glass electrodes filled with internal solution containing the following: 115 mM potassium methanesulfonate, 20 mM KCl, 10 mM HEPES, 2.5 mM MgCl_2_, 4 mM adenosine triphosphate disodium salt, 0.4 mM guanosine triphosphate trisodium salt, 10 mM sodium phosphocreatine, and 0.6 mM EGTA, pH 7.25, with KOH. For voltage-clamp recordings, the potassium was replaced by cesium.

All experiments and the analysis of data were performed in a blind manner. Recordings were performed using a MultiClamp 700B amplifier and Digidata 1440, and data were acquired and analyzed using Clampex 10.3 and Clampfit 10.3 (Molecular Devices).

### Imaging

The organotypic slices were fixed in 4% paraformaldehyde and 4% sucrose in PBS overnight. The slices were then cryoprotected in 30% sucrose in 0.1 M phosphate buffer (pH 7.4) for 2 h at room temperature followed by rapid freeze and thaw treatments. The slices were then stained with antibodies [γ-H2AX (Millipore, #05-636), GFP (MBL, #598), and secondary Alexa Fluor 647/488 dye-conjugated anti-mouse (Jackson ImmunoResearch) antibodies] in GDB buffer (0.1% gelatin, 0.5% TX-100, 450 mM NaCl, and 32% 0.1 M phosphate buffer, pH 7.4; McAllister, 2000). Two-photon and confocal microscopy were used for spine and γ-H2AX imaging, respectively. Two-photon images were captured using a LUMPlanN 60× (1.0 NA) objective. All secondary dendrites for each neuron were subjected to spine dimension and density analysis, and averaged values per dendritic segments were pooled. Scanimage software (Pologruto et al., 2003), allowed continuous acquisition of high-magnification images at 0.2-μm intervals at maximal resolution (512 × 512 pixels). Photomultiplier tube voltage settings were maintained at the same level for image collection for each cell. Registered projections of stacks collected were used to determine dendritic spine enumerations. Images of γ-H2AX in CA1 pyramidal neurons were obtained using confocal microscopy (Leica TCS SP8). Images were analyzed by MetaMorph and ImageJ software. All imaging and image analyses were performed in a blind manner.

### Statistical analysis

Results are reported as mean ± SEM. The statistical significance was evaluated by one- and two-way ANOVA with *post hoc* Tukey for multiple comparison. Mann–Whitney *U* test and Student’s *t* test were used for two group comparison. Statistical significance was set at *p* < 0.05 (Table 2).

## Results

### Basal excitatory synaptic transmission in shRNA-transfected neurons

**Figure 1.**
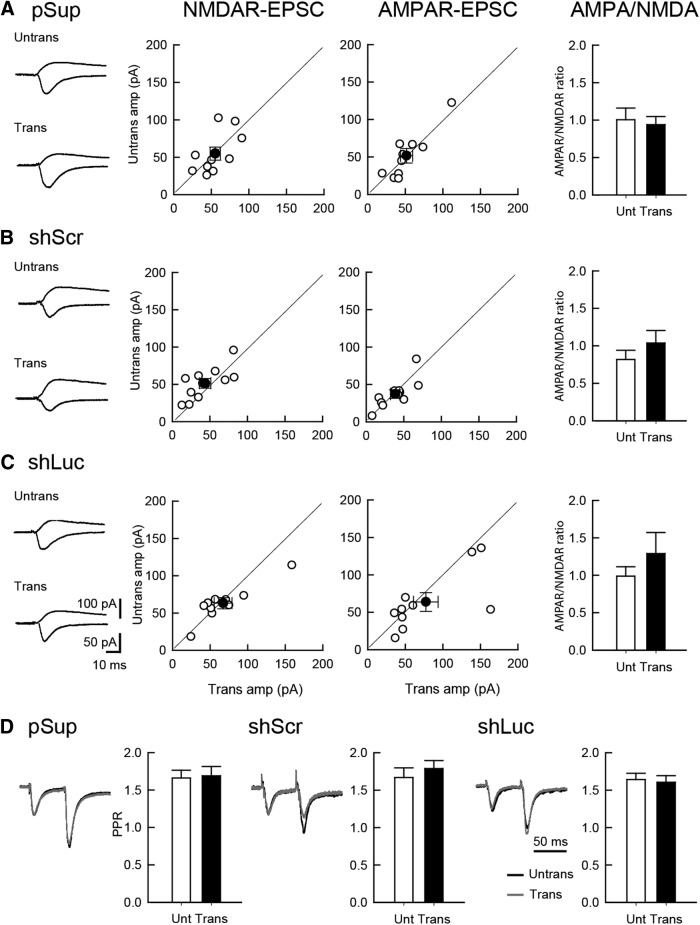
Comparable levels of excitatory synaptic transmission between shRNA-transfected and untransfected neurons. Effect of overexpression of three different plasmid transfections on excitatory synaptic transmission in hippocampal CA1 pyramidal cells. An empty vector (pSup; ***A***), shScr (***B***), and shLuc (***C***) were transfected together with pCAG-EGFP. Recordings were conducted 5 d following transfection. Left column, Sample EPSC traces mediated by AMPARs (downward) and NMDARs (upward) from pairs of transfected neurons (Trans) and neighboring untransfected neurons (Untrans). Stimulus artifacts were truncated. Middle columns, Scattered plots of NMDAR- (left) and AMPAR- (right) EPSC amplitude (amp). Each pair of transfected and neighboring untransfected cells are presented as open symbols while filled symbols indicate the mean. Right column, Bar graphs of AMPAR/NMDAR ratios. ***D***, PPR of AMPAR-EPSCs recorded from trans- and untransfected neurons, as indicated. Left, Sample traces. Normalized EPSCs to the first EPSC amplitude from trans- (gray) and untransfected neurons (black) are superimposed. Right, Summary graphs of PPR. The PPR was calculated by dividing the average amplitude of the second EPSC by that of the first EPSC. Number of cell pairs tested: pSup, 10 cells/6 mice; shLuc, 10/6; shScr, 11/6 (for AMPAR-EPSC, NMDAR-EPSC, and PPR, respectively).

To examine the physiologic effects of shRNAs on neuronal function, we prepared organotypic slice cultures from mouse hippocampi and biolistically transfected GFP with pSuper empty vector (pSup), luciferase shRNA (shLuc) and shScr. To test whether control shRNAs display abnormal off-target effect(s) in excitatory synaptic function, simultaneous whole-cell recordings of untransfected and transfected CA1 pyramidal neurons were performed. AMPAR- and NMDAR-mediated evoked EPSCs were measured by stimulating Shaffer collateral inputs (Fig. 1*A-C*). As expected, transfection of pSuper empty vector, shLuc or shScr exhibited no off-target effects on the amplitude of AMPAR- and NMDAR-EPSCs, as well as the AMPAR/NMDAR ratio, confirming previous results (Hoogenraad et al., 2010; Chen et al., 2014b). Paired-pulse ratio (PPR) measured by the AMPAR-EPSC response obtained by the double stimulation of Shaffer collateral inputs with a 50-ms interval exhibited comparable levels of facilitation in all transfected neurons regardless of the plasmids used, indicating that the transfection of these genes do not affect presynaptic release probability (Fig. 1*D*).

### Normal inhibitory synaptic transmission in shRNA-transfected neurons

**Figure 2.**
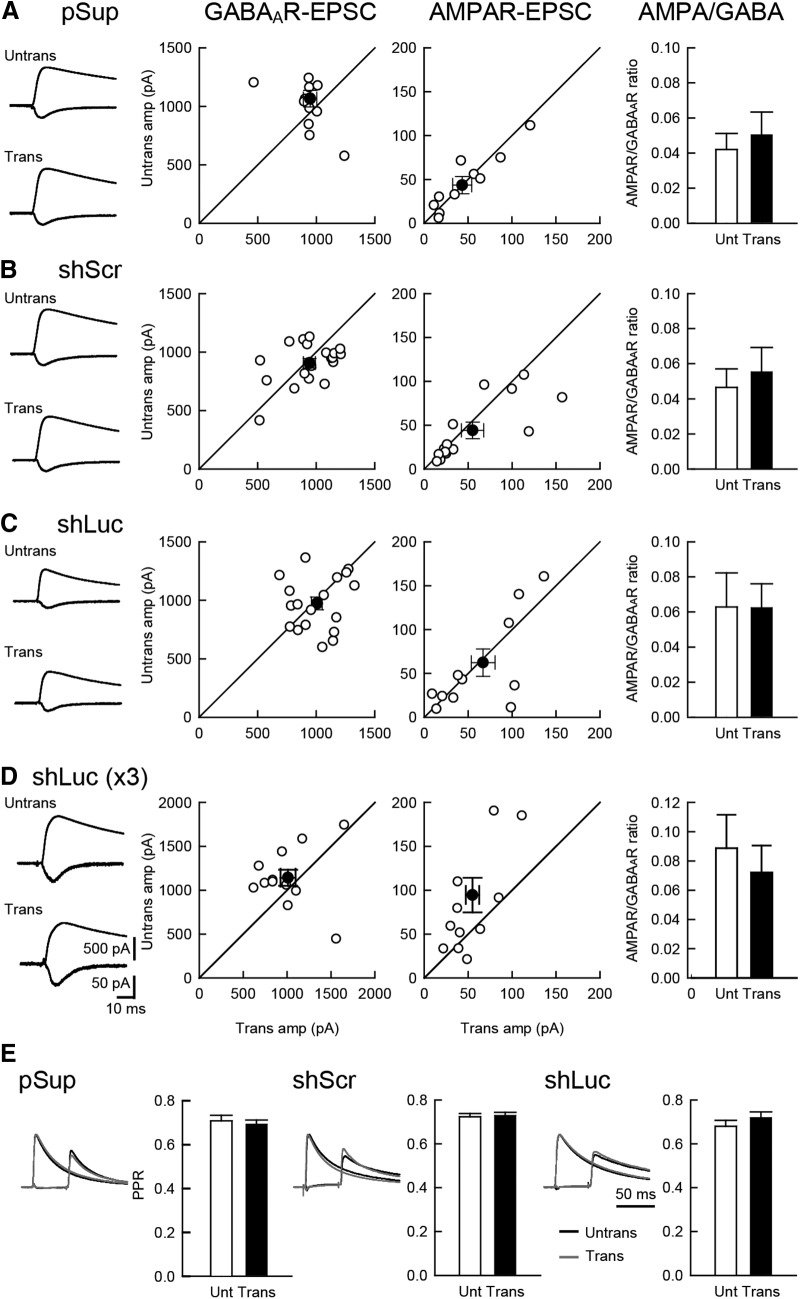
Comparable level of inhibitory synaptic transmission between shRNA-transfected and untransfected neurons. Effect of overexpression of the three different plasmid on excitatory and inhibitory synaptic transmission in hippocampal CA1 pyramidal cells. An empty vector (pSup; ***A***), shScr (***B***), shLuc (***C***) and shLuc x3 (triple the amount of shLuc) (***D***) were transfected together with pCAG-EGFP. Left column, Sample AMPAR-EPSC and GABA_A_R-IPSC traces mediated by AMPARs (downward) and GABA_A_Rs (upward) from pairs of transfected neurons (Trans) and neighboring untransfected neurons (Untrans). Stimulus artifacts were truncated. Middle columns, Scattered plots of GABA_A_R- (left) and AMPAR- (right) EPSC amplitude (amp). Each pair of transfected and neighboring untransfected cells are presented as open symbols while filled symbols indicate the mean. Right column, Bar graphs of AMPAR/GABA_A_R ratios. ***E***, PPR of GABA_A_R-IPSCs recorded from trans- and untransfected neurons, as indicated. Left, Sample traces. Normalized IPSCs to the first IPSC amplitude from trans- (gray) and untransfected neurons (black) were superimposed. The First GABA_A_R-IPSC overlaps with the second IPSC. Therefore, the first EPSC was cancelled by subtracting the traces receiving a single pulse from those receiving a paired pulse, both normalized to the first response. Right, Summary graphs of PPR. The PPR was calculated by dividing the average amplitude of the second IPSC by that of the first IPSC. Number of cell pairs: pSup, 13, 11, and 13 cells/7 mice (13, 11, 13/7); shLuc (18, 13, 18/8); shScr (20, 15, 20/8); shLuc x3 (13, 12/6), for GABA_A_R-IPSC, AMPAR-EPSC, and PPR, respectively.

Next, we examined the effects of shRNAs on inhibitory synaptic transmission and the balance of excitatory and inhibitory transmission. Neither GABA_A_R-mediated IPSC nor the ratio of AMPAR and GABA_A_R responses displayed significant changes following transfection of any of the three plasmids (Fig. 2*A–C*). Paired-pulse stimulation of inhibitory inputs exhibited comparable levels of depression in all transfected neurons, suggesting that the transfection of these genes do not have any effects on presynaptic release probability (Fig. 2*E*).

In contrast to our results, Alvarez et al. (2006) reported that shLuc displayed reduced inhibitory and excitatory synaptic transmission in organotypic hippocampal CA1 neurons. The dosage of shRNAs, reported to contribute to off-target effects (Jackson et al., 2006a), may account for this discrepancy as Alvarez et al. (2006) used a 1.3-fold larger amount of shRNA than used in the present study. To test this possibility, we increased the shLuc plasmid concentration three-fold and measured IPSCs and AMPAR-EPSCs (Fig. 2*D*). A three-fold increase of shLuc did not significantly reduce synaptic transmission, indicating that a potential dosage-dependent off-target effect of shLuc is small in synaptic function as measured here and most likely is not the primary reason for the differences in results between those reported here and by Alvarez et al. (2006).

### Normal dendritic morphology in shRNA-transfected neurons

**Figure 3.**
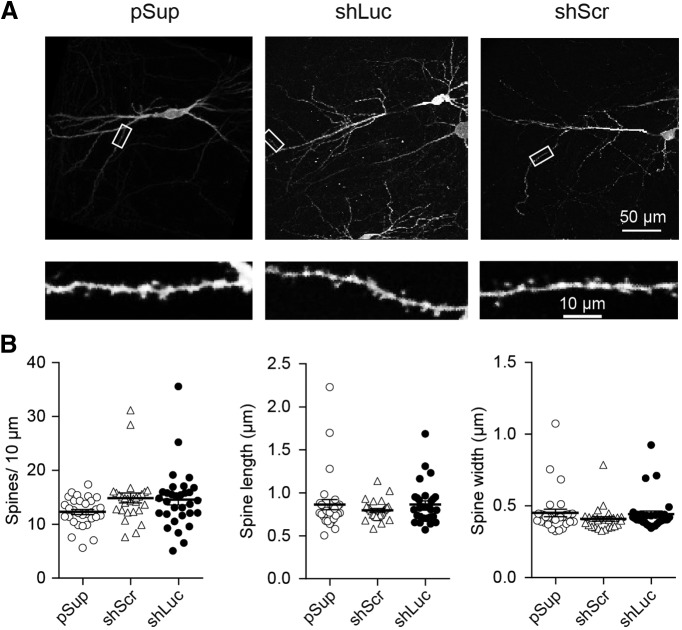
Comparable level of dendritic structure between different shRNA-transfected neurons. Effect of overexpression of three different plasmid transfections on dendritic morphology in hippocampal CA1 pyramidal neurons. ***A***, Low (top) and high (bottom) magnification images obtained from empty vector, pSup (left), shLuc (middle), and shScr (right) transfected neurons. Each plasmid was cotransfected with pCAG-EGFP. Fixed slices were immunostained against GFP and neuronal images were obtained by two-photon microscopy. ***B***, Scatter plots of spine length (left), width (middle), and density (right). Error bars indicate SEMs. Note that none of these parameters displayed statistical significance by gene transfection. Number of dendritic segments/cells/mice: pSup, 31/5/5; shLuc, 31/5/5; and shScr, 28/5/5.

We next addressed the effects of shRNAs on neuronal morphology. We overexpressed shRNAs together with EGFP. The transfected neurons were immunostained against GFP followed by imaging using two-photon microscopy (Fig. 3). The dendritic morphology, including spine density and dimension, was found to be unchanged by overexpression of the two shRNAs. Overall, we did not identify off-target effects of control shRNAs in synaptic function and structure.

### Reduced membrane excitability and voltage-gated ion channel function in shLuc-transfected CA1 pyramidal neurons

**Figure 4.**
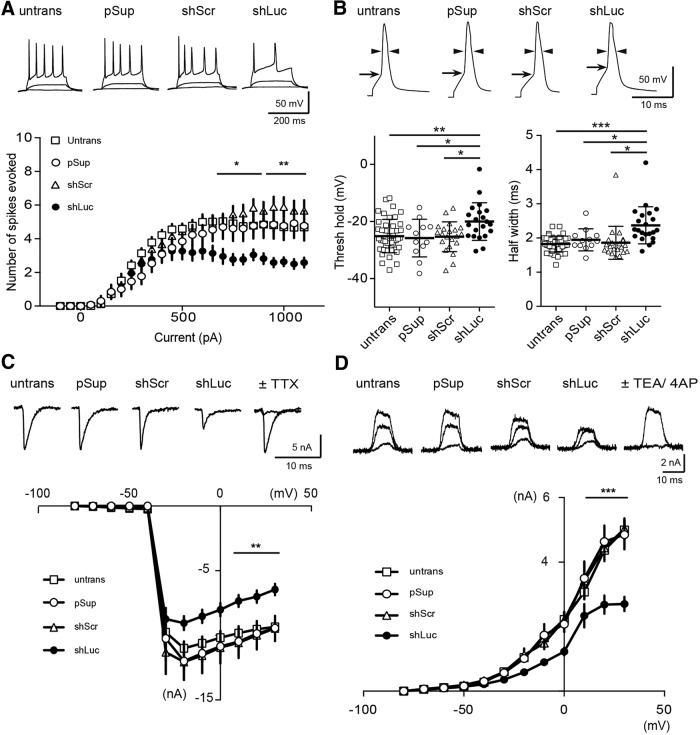
Reduced membrane excitabilities in shLuc-transfected CA1 pyramidal neurons. ***A***, Effect of shRNA overexpression on neuronal excitability. An empty vector (pSup), shLuc, or shScr was transfected together with pCAG-EGFP. Top, Sample traces from untransfected and transfected CA1 pyramidal neurons in organotypic hippocampal slice cultures. The superimposed traces were elicited by current injections of 0, 100, and 500 pA for 200 ms. Bottom, Summary graph of the frequency of APs in untransfected and transfected neurons. The input–output relationship [number of spikes elicited versus amount of current injection (200-ms duration)] was plotted for untransfected and transfected neurons. Neurons were held at resting membrane potentials (−56.8 mV ± 0.57, *n* = 100 cells, 9 mice). Number of cells tested: untrans, 45 cells from eight mice (45/8); pSup, 13/7; shScr, 20/5; and shLuc, 22/9. ***B***, Effect of shRNA overexpression on AP kinetics. Top, Sample traces from CA1 pyramidal neurons untransfected and transfected with the three different plasmids. Single APs were induced by current injection (100 pA for 4 ms) and threshold (horizontal arrows) and half width of AP determined by vertical double arrow heads in trans- and untransfected neurons were measured. Bottom, Summary graph of the threshold (left) and half width (right) of single AP in untransfected and transfected neurons. Number of cells tested: untrans, 45 cells/8 mice (45/8); pSup, 13/7; shScr, 20/5; shLuc, 22/9. ***C***, top, Sample traces of sodium currents recorded from untransfected and transfected CA1 pyramidal neurons. Note that these currents were completely blocked by TTX (right). Bottom, Summary graph of the sodium currents in untransfected and transfected neurons. Neurons were voltage-clamped at –80 mV and depolarized from –80 to 30 mV (10-ms duration). Number of cells: untrans, 16 cells/5 mice; pSup, 10/5; shScr, 10/5; shLuc, 14/6. ***D***, top, Sample traces of potassium currents recorded from untransfected and transfected CA1 pyramidal neurons. Note that these upward currents were completely blocked by TEA and 4-AP (right). Bottom, Summary graph of the potassium currents in untransfected and transfected neurons. Neurons were voltage-clamped at –80 mV and depolarized from –80 to 30 mV (10-ms duration). Number of cells tested: untrans, 15 cells/4 mice; pSup, 13/3; shScr, 7/3; shLuc, 9/3.

To examine the effects of shRNAs on membrane properties, neurons transfected with shRNAs and EGFP were compared to untransfected neurons with respect to action potentials (APs) and basic membrane properties (Fig. 4; Table 1). To our great surprise, CA1 neurons transfected with shLuc exhibited a reduced number of APs compared to untransfected control neurons (Fig. 4*A*). The transfection of pSup did not cause any abnormal excitability, indicating that this vector backbone is not the cause of the reduced excitability in shLuc-transfected neurons.

**Table 1.** The basic membrane properties of untransfected and gene transfected hippocampal CA1 neurons

Cell types	Peak amplitude of AP (mV)	Resting membrane potential (mV)	Input resistance (MΩ)	Series resistance (MΩ)	Number of cells/mice
untrans	106.1 ± 1.331	−55.87 ± 0.900	105.6 ± 8.452	8.127 ± 0.2768	45/8
pSup	108.5 ± 2.452	−58.51 ± 1.604	122.9 ± 18.62	8.869 ± 0.6093	13/7
shScr	109.3 ± 1.456	−57.47 ± 1.104	143.1 ± 18.03	8.456 ± 0.3902	20/5
shLuc	106.0 ± 2.224	−57.07 ± 1.054	131.0 ± 11.29	9.349 ± 0.3110	22/9
Statistics *p* value	shLuc vs shScr 0.7606	shLuc vs shScr >0.9999	shLuc vs shScr 0.9893	shLuc vs shScr 0.4875	
	shLuc vs pSup 0.9657	shLuc vs pSup 0.9777	shLuc vs pSup 0.9996	shLuc vs pSup 0.9755	
	shLuc vs untrans >0.9999	shLuc vs untrans 0.9604	shLuc vs untrans 0.567	shLuc vs untrans 0.0681	
	shScr vs untrans 0.6744	shScr vs pSup 0.9958	shScr vs pSup 0.9434	shScr vs pSup 0.988	
	shScr vs pSup >0.9999	shScr vs untrans 0.8504	shScr vs untrans 0.1487	shScr vs untrans 0.9813	
	pSup vs untrans 0.9603	pSup vs untrans 0.5991	pSup vs untrans 0.9558	pSup vs untrans 0.7507	

Note that all of these parameters were not statistically different between four cell groups. Statistics were done by one-way ANOVA with *post hoc* Tukey.

**Table 2.** Statistical table

Graph	Data structure	Type of test	Dataset	*p* value
Fig. 1*A*	Nonparametric	Mann-Whitney test	AMPAR-EPSC	0.97
			NMDAR-EPSC	0.51
			AMPAR/NMDAR ratio	0.97
Fig. 1*B*	Nonparametric	Mann-Whitney test	AMPAR-EPSC	0.69
			NMDAR-EPSC	0.47
			A/N ratio	0.33
Fig. 1*C*	Nonparametric	Mann-Whitney test	AMPAR-EPSC	0.79
			NMDAR-EPSC	0.79
			A/N ratio	0.47
Fig. 1*D*	Parametric	Student’s *t* test	PPR of pSup	0.86
			PPR of shScr	0.48
			PPR of shLuc	0.76

Fig. 2*A*	Nonparametric	Mann-Whitney test	GABA_A_R-IPSC	0.21
			AMPAR-EPSC	1
			AMPAR/GABA_A_R ratio	0.55
Fig. 2*B*	Nonparametric	Mann-Whitney test	GABA_A_R-IPSC	0.43
			AMPAR-EPSC	0.41
			A/G ratio	0.56
Fig. 2*C*	Nonparametric	Mann-Whitney test	GABA_A_R-IPSC	0.69
			AMPAR-EPSC	0.92
			A/G ratio	0.89
Fig. 2*D*	Nonparametric	Mann-Whitney test	GABA_A_R-IPSC	0.15
			AMPAR-EPSC	0.24
			A/G ratio	0.58
Fig. 2*E*	Parametric	Student’s *t* test	PPR of pSup	0.63
			PPR of shScr	0.74
			PPR of shLuc	0.31
Fig. 3*B*	Parametric	One-way ANOVA *post hoc* Tukey	Spine density: pSup vs shLuc	0.45
			pSup vs shScr	0.09
			shScr vs shLuc	0.65
			Spine length: pSup vs shLuc	0.99
			pSup vs shScr	0.68
			shScr vs shLuc	0.63
			Spine width: pSup vs shLuc	0.96
			pSup vs shScr	0.37
			shScr vs shLuc	0.53
Fig. 4*A*	Parametric	Two-way ANOVA *post hoc* Tukey	−100 pA	
			untrans vs shLuc	>0.9999
			untrans vs shScr	>0.9999
			untrans vs pSup	>0.9999
			shLuc vs shScr	>0.9999
			shLuc vs pSup	>0.9999
			shScr vs pSup	>0.9999
			−50 pA	
			untrans vs shLuc	>0.9999
			untrans vs shScr	>0.9999
			untrans vs pSup	>0.9999
			shLuc vs shScr	>0.9999
			shLuc vs pSup	>0.9999
			shScr vs pSup	>0.9999
			0 pA	
			untrans vs shLuc	>0.9999
			untrans vs shScr	>0.9999
			untrans vs pSup	>0.9999
			shLuc vs shScr	>0.9999
			shLuc vs pSup	>0.9999
			shScr vs pSup	>0.9999
			50 pA	
			untrans vs shLuc	>0.9999
			untrans vs shScr	>0.9999
			untrans vs pSup	0.9993
			shLuc vs shScr	>0.9999
			shLuc vs pSup	0.9995
			shScr vs pSup	0.9996
			100 pA	
			untrans vs shLuc	0.9997
			untrans vs shScr	>0.9999
			untrans vs pSup	0.9923
			shLuc vs shScr	0.9995
			shLuc vs pSup	0.9978
			shScr vs pSup	0.9928

			150 pA	
			untrans vs shLuc	0.9909
			untrans vs shScr	>0.9999
			untrans vs pSup	0.9964
			shLuc vs shScr	0.9959
			shLuc vs pSup	>0.9999
			shScr vs pSup	0.9982
			200 pA	
			untrans vs shLuc	0.9818
			untrans vs shScr	>0.9999
			untrans vs pSup	0.8433
			shLuc vs shScr	0.9915
			shLuc vs pSup	0.9674
			shScr vs pSup	0.8983
			250 pA	
			untrans vs shLuc	0.7984
			untrans vs shScr	0.9421
			untrans vs pSup	0.4089
			shLuc vs shScr	0.9945
			shLuc vs pSup	0.8912
			shScr vs pSup	0.8019
			300 pA	
			untrans vs shLuc	0.6045
			untrans vs shScr	0.8776
			untrans vs pSup	0.124
			shLuc vs shScr	0.9847
			shLuc vs pSup	0.714
			shScr vs pSup	0.5448
			350 pA	
			untrans vs shLuc	0.2035
			untrans vs shScr	0.8942
			untrans vs pSup	0.1894
			shLuc vs shScr	0.7588
			shLuc vs pSup	0.9865
			shScr vs pSup	0.6361
			400 pA	
			untrans vs shLuc	0.1775
			untrans vs shScr	0.7432
			untrans vs pSup	0.2523
			shLuc vs shScr	0.8662
			shLuc vs pSup	0.9987
			shScr vs pSup	0.844
			450 pA	
			untrans vs shLuc	0.0599
			untrans vs shScr	0.8942
			untrans vs pSup	0.2917
			shLuc vs shScr	0.4825
			shLuc vs pSup	0.9928
			shScr vs pSup	0.7592
			500 pA	
			untrans vs shLuc	0.0477
			untrans vs shScr	0.9921
			untrans vs pSup	0.6175
			shLuc vs shScr	0.2411
			shLuc vs pSup	0.8394
			shScr vs pSup	0.8383
			550 pA	
			untrans vs shLuc	0.0101
			untrans vs shScr	0.9971
			untrans vs pSup	0.8145
			shLuc vs shScr	0.0831
			shLuc vs pSup	0.4248
			shScr vs pSup	0.9297

			600 pA	
			untrans vs shLuc	0.0043
			untrans vs shScr	>0.9999
			untrans vs pSup	0.68
			shLuc vs shScr	0.0299
			shLuc vs pSup	0.437
			shScr vs pSup	0.7694
			650 pA	
			untrans vs shLuc	0.0014
			untrans vs shScr	0.995
			untrans vs pSup	0.8638
			shLuc vs shScr	0.0266
			shLuc vs pSup	0.176
			shScr vs pSup	0.9617
			700 pA	
			untrans vs shLuc	0.0003
			untrans vs shScr	0.9998
			untrans vs pSup	0.9417
			shLuc vs shScr	0.0061
			shLuc vs pSup	0.0571
			shScr vs pSup	0.9735
			750 pA	
			untrans vs shLuc	0.0002
			untrans vs shScr	0.7664
			untrans vs pSup	0.9643
			shLuc vs shScr	0.0001
			shLuc vs pSup	0.038
			shScr vs pSup	0.6549
			800 pA	
			untrans vs shLuc	0.0007
			untrans vs shScr	0.5283
			untrans vs pSup	0.9959
			shLuc vs shScr	<0.0001
			shLuc vs pSup	0.038
			shScr vs pSup	0.6058
			850 pA	
			untrans vs shLuc	0.0041
			untrans vs shScr	0.2197
			untrans vs pSup	>0.9999
			shLuc vs shScr	<0.0001
			shLuc vs pSup	0.0601
			shScr vs pSup	0.4478
			900 pA	
			untrans vs shLuc	0.0005
			untrans vs shScr	0.4401
			untrans vs pSup	>0.9999
			shLuc vs shScr	<0.0001
			shLuc vs pSup	0.0174
			shScr vs pSup	0.6624
			950 pA	
			untrans vs shLuc	0.0001
			untrans vs shScr	0.1954
			untrans vs pSup	0.9999
			shLuc vs shScr	<0.0001
			shLuc vs pSup	0.006
			shScr vs pSup	0.466
			1000 pA	
			untrans vs shLuc	0.0004
			untrans vs shScr	0.1282
			untrans vs pSup	0.9992
			shLuc vs shScr	<0.0001
			shLuc vs pSup	0.0108
			shScr vs pSup	0.4014

			1050 pA	
			untrans vs shLuc	0.0001
			untrans vs shScr	0.2644
			untrans vs pSup	0.9762
			shLuc vs shScr	<0.0001
			shLuc vs pSup	0.0025
			shScr vs pSup	0.728
			1100 pA	
			untrans vs shLuc	0.0003
			untrans vs shScr	0.2803
			untrans vs pSup	0.999
			shLuc vs shScr	<0.0001
			shLuc vs pSup	0.0087
			shScr vs pSup	0.5944
Fig. 4*B*	Parametric	One-way ANOVA *post hoc* Tukey	Threshhold:	
			shLuc vs shScr	0.0249
			shLuc vs pSup	0.0421
			shLuc vs untrans	0.0094
			shScr vs pSup	0.9958
			shScr vs untrans	0.9991
			pSup vs untrans	0.9837
			Half width**:**	
			shLuc vs shScr	0.0002
			shLuc vs pSup	0.0165
			shLuc vs untrans	<0.0001
			shScr vs pSup	0.9231
			shScr vs untrans	0.9846
			pSup vs untrans	0.7637
Fig. 4*C*	Parametric	Two-way ANOVA *post hoc* Tukey	-80 mV	
			shLuc vs shScr	>0.9999
			shLuc vs pSup	>0.9999
			shLuc vs untrans	>0.9999
			shScr vs pSup	>0.9999
			shScr vs untrans	>0.9999
			pSup vs untrans	>0.9999
			-70 mV	
			shLuc vs shScr	>0.9999
			shLuc vs pSup	0.9998
			shLuc vs untrans	>0.9999
			shScr vs pSup	>0.9999
			shScr vs untrans	>0.9999
			pSup vs untrans	>0.9999
			-60 mV	
			shLuc vs shScr	>0.9999
			shLuc vs pSup	0.9982
			shLuc vs untrans	0.9994
			shScr vs pSup	0.9995
			shScr vs untrans	>0.9999
			pSup vs untrans	0.9999
			−50 mV	
			shLuc vs shScr	0.9996
			shLuc vs pSup	0.9956
			shLuc vs untrans	0.998
			shScr vs pSup	0.9992
			shScr vs untrans	0.9999
			pSup vs untrans	0.9999
			−40 mV	
			shLuc vs shScr	0.999
			shLuc vs pSup	0.2815
			shLuc vs untrans	0.9959
			shScr vs pSup	0.3548
			shScr vs untrans	0.9999
			pSup vs untrans	0.3237

			−30 mV	
			shLuc vs shScr	0.0008
			shLuc vs pSup	0.0272
			shLuc vs untrans	0.0755
			shScr vs pSup	0.7329
			shScr vs untrans	0.315
			pSup vs untrans	0.9295
			−20 mV	
			shLuc vs shScr	0.0007
			shLuc vs pSup	0.0009
			shLuc vs untrans	0.0235
			shScr vs pSup	0.9999
			shScr vs untrans	0.5293
			pSup vs untrans	0.5778
			−10 mV	
			shLuc vs shScr	0.0007
			shLuc vs pSup	0.0013
			shLuc vs untrans	0.0183
			shScr vs pSup	0.9983
			shScr vs untrans	0.5872
			pSup vs untrans	0.7019
			0 mV	
			shLuc vs shScr	0.001
			shLuc vs pSup	0.0014
			shLuc vs untrans	0.015
			shScr vs pSup	0.9998
			shScr vs untrans	0.7013
			pSup vs untrans	0.7536
			10 mV	
			shLuc vs shScr	0.0005
			shLuc vs pSup	0.0008
			shLuc vs untrans	0.0083
			shScr vs pSup	0.9996
			shScr vs untrans	0.6986
			pSup vs untrans	0.7654
			20 mV	
			shLuc vs shScr	0.0011
			shLuc vs pSup	0.0017
			shLuc vs untrans	0.0064
			shScr vs pSup	0.9994
			shScr vs untrans	0.856
			pSup vs untrans	0.9091
			30 mV	
			shLuc vs shScr	0.0015
			shLuc vs pSup	0.0017
			shLuc vs untrans	0.0042
			shScr vs pSup	>0.9999
			shScr vs untrans	0.9547
			pSup vs untrans	0.9613
Fig. 4*D*	Parametric	Two-way ANOVA *post hoc* Tukey	−80 mV	
			shLuc vs shScr	>0.9999
			shLuc vs pSup	>0.9999
			shLuc vs untrans	>0.9999
			shScr vs pSup	>0.9999
			shScr vs untrans	>0.9999
			pSup vs untrans	>0.9999
			−70 mV	
			shLuc vs shScr	>0.9999
			shLuc vs pSup	0.9998
			shLuc vs untrans	>0.9999
			shScr vs pSup	>0.9999
			shScr vs untrans	>0.9999
			pSup vs untrans	>0.9999

			−60 mV	
			shLuc vs shScr	>0.9999
			shLuc vs pSup	0.9982
			shLuc vs untrans	0.9994
			shScr vs pSup	0.9995
			shScr vs untrans	>0.9999
			pSup vs untrans	0.9999
			−50 mV	
			shLuc vs shScr	0.9996
			shLuc vs pSup	0.9956
			shLuc vs untrans	0.998
			shScr vs pSup	0.9992
			shScr vs untrans	0.9999
			pSup vs untrans	0.9999
			−40 mV	
			shLuc vs shScr	0.999
			shLuc vs pSup	0.2815
			shLuc vs untrans	0.9959
			shScr vs pSup	0.3548
			shScr vs untrans	0.9999
			pSup vs untrans	0.3237
			−30 mV	
			shLuc vs shScr	0.0008
			shLuc vs pSup	0.0272
			shLuc vs untrans	0.0755
			shScr vs pSup	0.7329
			shScr vs untrans	0.315
			pSup vs untrans	0.9295
			−20 mV	
			shLuc vs shScr	0.0007
			shLuc vs pSup	0.0009
			shLuc vs untrans	0.0235
			shScr vs pSup	0.9999
			shScr vs untrans	0.5293
			pSup vs untrans	0.5778
			−10 mV	
			shLuc vs shScr	0.0007
			shLuc vs pSup	0.0013
			shLuc vs untrans	0.0183
			shScr vs pSup	0.9983
			shScr vs untrans	0.5872
			pSup vs untrans	0.7019
			0 mV	
			shLuc vs shScr	0.001
			shLuc vs pSup	0.0014
			shLuc vs untrans	0.015
			shScr vs pSup	0.9998
			shScr vs untrans	0.7013
			pSup vs untrans	0.7536
			10 mV	
			shLuc vs shScr	0.0005
			shLuc vs pSup	0.0008
			shLuc vs untrans	0.0083
			shScr vs pSup	0.9996
			shScr vs untrans	0.6986
			pSup vs untrans	0.7654
			20 mV	
			shLuc vs shScr	0.0011
			shLuc vs pSup	0.0017
			shLuc vs untrans	0.0064
			shScr vs pSup	0.9994
			shScr vs untrans	0.856
			pSup vs untrans	0.9091

			30 mV	
			shLuc vs shScr	0.0015
			shLuc vs pSup	0.0017
			shLuc vs untrans	0.0042
			shScr vs pSup	>0.9999
			shScr vs untrans	0.9547
			pSup vs untrans	0.9613

Fig. 5*A*	Parametric	Student’s *t* test	Ctrl vs CPT	<0.00001
Fig. 5*B*	Parametric	One-way ANOVA *post hoc* Tukey	shLuc vs shScr	0.8937
			shLuc vs pSup	0.885
			shScr vs pSup	0.9996

To further test whether shLuc changed the threshold and kinetics of APs, we compared the onset, threshold, amplitude and half-width of APs in untransfected and transfected neurons (Fig. 4*B*; Table 1). We found that APs in shLuc-transfected neurons exhibited increased half-width (Fig. 4*B*, lower right) and decrease of threshold (Fig. 4*B*, lower left) without changing the amplitude of AP (Table 1). The basic membrane properties, including resting membrane potential and series and input resistance, remained unchanged, indicating an off-target effect of shLuc on voltage-gated ion channel function (Table 1). Taken together, our results strongly suggest that shLuc has off-target effect(s) on membrane excitability and AP kinetics in CA1 pyramidal neurons.

Given these striking results, we next addressed the underlying mechanism of the off-target effect of shLuc on membrane excitability and APs. Whole-cell voltage clamp recordings were performed in CA1 pyramidal neurons and the current−voltage (I-V) relationship of TTX-sensitive sodium (Na^+^) channel current was measured. Importantly, Na^+^ current maximum amplitude between −20 and +30 mV was markedly reduced in shLuc-transfected compared to untransfected neurons (Fig. 4*C*, closed circles). We then tested whether voltage-dependent potassium (K^+^) currents were altered by shLuc transfection. The potassium channel currents, sensitive to the broad potassium channel blockers, TEA, and 4-AP, were also markedly reduced in shLuc-transfected neurons compared with other neurons (Fig. 4*D*, closed circle). These results indicate that shLuc has off-target effects on voltage-gated sodium and potassium channels.

### Normal H2A-X phosphorylation in shRNA-transfected neurons

**Figure 5.**
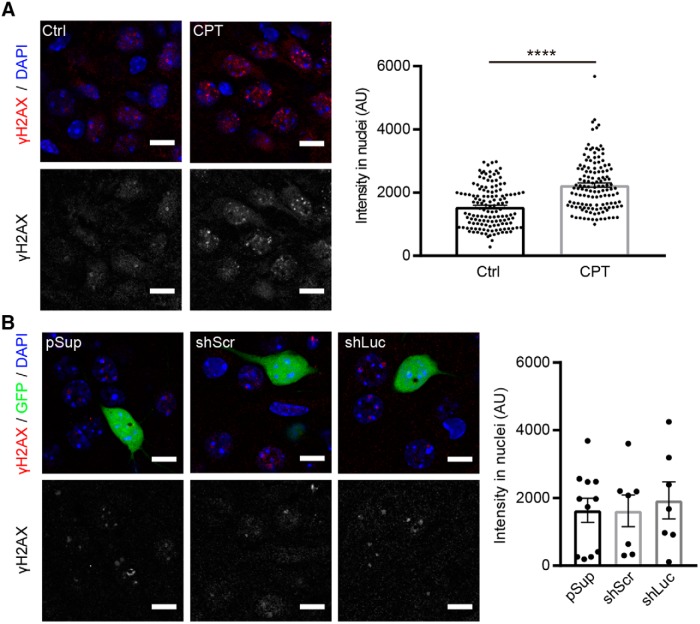
Comparable levels of H2A-X phosphorylation in neurons transfected with different shRNA vectors. γ-H2AX immunoreactivity in shRNA-transfected hippocampal CA1 neurons. ***A***, left, Representative confocal images of immunofluorescence staining against γ-H2AX (red) and DAPI (blue) in CA1 pyramidal neurons. Right, Quantification of γ-H2AX signal intensity in nuclei. Treatment with 10 μM camptothecin (CPT) for 6 h increased the signal of γ-H2AX in nuclei of neurons in organotypic slice cultures, confirming the specificity of the anti-γ-H2AX antibody. AU, arbitrary units. Number of cells/mice: mock control (Ctrl), 140/3; CPT, 133/3. ***B***, left, Representative confocal images of immunofluorescence staining against γ-H2AX (red), GFP epifluorescence (green) and DAPI (blue) in pSup-, shLuc-, and shScr-transfected neurons. Right, Quantification of γ-H2AX signal intensity in nuclei. All transfected neurons exhibited comparable levels of γ-H2AX signal. Number of cells/mice: pSup, 11/3; shScr, 7/4; shLuc, 7/3. Scale bars: 10 μm.

Lastly, we addressed whether the reduced membrane excitability in shLuc-transfected neurons is due to potential cellular stress or damage to cellular functions. To test this hypothesis, we examined the level of the phosphorylation of histone H2A.X. H2A.X phosphorylation at serine 139 (γ-H2AX) is a routinely used biomarker to detect DNA damage and DNA replication stress (Mah et al., 2010). γ-H2AX is greatly increased in neurons by seizure insult (Crowe et al., 2011) and camptothecin, an inhibitor of the DNA enzyme topoisomerase I (Mah et al., 2010; Fig. 5*A*). Importantly, the level of γ-H2AX in shLuc-transfected neurons was comparable to that of pSup- and shScr-transfected neurons, suggesting that transfection of shLuc does not alter the overall health of the neurons (Fig. 5*B*). In summary, we conclude that shLuc exhibits off-target effects on membrane excitability without changing synaptic transmission, dendritic morphology or cellular health.

## Discussion

RNAi is a powerful approach to validate the function of target genes both *in vitro* and *in vivo*. In light of repeated indications that shRNAs may have off-target effects (Cullen, 2006), their effects should be carefully studied. Here, we found that shLuc transfection increased AP threshold and half-width in addition to reducing the number of APs. shLuc also reduced TTX-sensitive sodium and TEA- and 4AP-sensitive potassium currents that contribute to shaping APs. In contrast to its effects on these currents, shLuc failed to alter basic membrane properties, suggesting that shLuc does not exhibit off-target effects on pumps (e.g., Na+/K^+^-ATPase) and other channels (e.g., leak channels) that contribute to maintaining resting membrane potential.

Is there any possibilities that shLuc targets the genes regulating the function of voltage-gated ion channels? A BLAST search against shLuc guide sequence (19 base-pairs: TCGAAGTATTCCGCGTACG), the strand incorporated into RNA-induced silencing complex, displayed seven genes with low homology (11–12 bp match, 58–63% homology), including *Dnah11* (dynein axonemal heavy chain 11, XM_017314949.1), *Plpp5* (phospholipid phosphatase 5, NM_001293703.1), *Lpcat2* (lysophosphatidylcholine acyltransferase 2, XM_006531052.2), *Asb7* (Ankyrin repeat and SOCS box-containing 7, XM_006540568.3), *Ipo8* (importin 8, XR_001785142.1), *Dis3* (DIS3 homolog exosome endoribonuclease and 3´->5´ exoribonuclease, XM_006519602.3), *Fbcl20* (F-box and leucine-rich repeat protein 20, XM_006534299.3). Based on the homology, it is difficult to consider these genes as the potential target of shLuc and none of these have been known as the regulators of Na^+^ and K^+^ channels.

Alvarez et al. (2006) reported that shLuc caused a lack of dendritic spines and reduced excitatory and inhibitory synaptic transmission in hippocampal pyramidal neurons. However, we were unable to confirm their observations. As described in our results, neurons transfected with shLuc display normal synaptic transmission and dendritic spine structure compared with untransfected and other genes transfected neurons. Our results are also supported by other studies demonstrating normal excitatory synaptic transmission, long-term potentiation and dendritic morphology in shLuc-transfected neurons (Hoogenraad et al., 2010; Wakita et al., 2011; Chen et al., 2014a,b). Since Alvarez et al. (2006) used the same experimental approach as ours (Stoppini et al., 1991), this discrepancy may be due to differences in precise details of the experimental procedures, including the vector backbone and the methods of gene transfection in organotypic slice cultures. For example, Alvarez et al. (2006) used U6 promoter to synthesize shLuc but pSuper vector consists with H1 promoter. Both H1 and U6 promoters are member of the type III class of Pol III RNA promoters, but it is possible that these two different promoters cause the distinct off-target effects in neurons (Mäkinen et al., 2006). However, regardless of this diverging result, it is clear that shLuc exhibits off-target effects on ion channel or synaptic function, discouraging the usage of shLuc as a control shRNA, at least in hippocampal CA1 pyramidal cells.

While comprehensive guidelines for the RNAi approach have been described (2003), detailed studies of off-target effects of control shRNAs are very limited. Thus, our comparative study on shRNAs in pyramidal neurons is an important consideration for future RNAi experiments. Of note, gene expression is differentially regulated by cell-type, cellular activity and development. Therefore, careful evaluation is required for shRNA constructs, including nonsilencing controls. However, we consider that shScr is currently the best validated control for the RNAi approach in mammalian cells including neurons.

## Synthesis

Reviewing Editor: Christophe Bernard, INSERM & Institut de Neurosciences des Systèmes Decisions are customarily a result of the Reviewing Editor and the peer reviewers coming together and discussing their recommendations until a consensus is reached. When revisions are invited, a fact-based synthesis statement explaining their decision and outlining what is needed to prepare a revision will be listed below. The following reviewer(s) agreed to reveal their identity: Weinan Sun.

Both reviewers noted the high quality of the data presented here.

The result is very important for the community, and we agree that at this stage, it may not be necessary to provide a mechanism. However, it is essential for this kind of study not to add confusion to the field.

1. We agree that it is important for you to test if the cells transfected with shLuc are as healthy as controls. Knowing that shLuc damages or not the cells would be really useful.

2. The lack of consistency with Alvarez et al., 2006 raises important issues. Since you propose that dosage may be an explanation, would it be possible to perform a dose-response curve?

Minor

Methods section:

“35 C{degree sign}” should be “35 {degree sign}C”.

post hoc “Turkey” should be “Tukey”.

The n value for statistical analysis of Figure 3 needs to be specified. Authors refer to 5 cells per 5 mice per group, however the number of dots represented in the graphs seems a lot larger.

Figure 4D should indicate TEA not TAE.

The statistical analysis described in table 1 should be included in the statistics table with p values.

Table 1 should read shLuc not shLuci.

## References

[B1] (2003) Whither RNAi? Nat Cell Biol 5:489–490. 12776118 10.1038/ncb0603-490

[B2] Alvarez VA, Ridenour DA, Sabatini BL (2006) Retraction of synapses and dendritic spines induced by off-target effects of RNA interference. J Neurosci 26:7820–7825. 10.1523/JNEUROSCI.1957-06.2006 16870727 PMC6674211

[B3] Birmingham A, Anderson EM, Reynolds A, Ilsley-Tyree D, Leake D, Fedorov Y, Baskerville S, Maksimova E, Robinson K, Karpilow J, Marshall WS, Khvorova A (2006) 3' UTR seed matches, but not overall identity, are associated with RNAi off-targets. Nat Methods 3:199–204. 10.1038/nmeth854 16489337

[B4] Chen Y, Wang Y, Modrusan Z, Sheng M, Kaminker JS (2014a) Regulation of neuronal gene expression and survival by basal NMDA receptor activity: a role for histone deacetylase 4. J Neurosci 34:15327–15339. 25392500 10.1523/JNEUROSCI.0569-14.2014 PMC4228135

[B5] Chen Y, Wang Y, Erturk A, Kallop D, Jiang Z, Weimer RM, Kaminker J, Sheng M (2014b) Activity-induced Nr4a1 regulates spine density and distribution pattern of excitatory synapses in pyramidal neurons. Neuron 83:431–443. 24976215 10.1016/j.neuron.2014.05.027

[B6] Crowe SL, Tsukerman S, Gale K, Jorgensen TJ, Kondratyev AD (2011) Phosphorylation of histone H2A.X as an early marker of neuronal endangerment following seizures in the adult rat brain. J Neurosci 31:7648–7656. 10.1523/JNEUROSCI.0092-11.2011 21613478 PMC3118469

[B7] Cullen BR (2006) Enhancing and confirming the specificity of RNAi experiments. Nat Methods 3:677–681. 10.1038/nmeth913 16929311

[B8] Fire A, Xu S, Montgomery MK, Kostas SA, Driver SE, Mello CC (1998) Potent and specific genetic interference by double-stranded RNA in *Caenorhabditis elegans* . Nature 391:806–811. 10.1038/35888 9486653

[B9] Futai K, Doty CD, Baek B, Ryu J, Sheng M (2013) Specific trans-synaptic interaction with inhibitory interneuronal neurexin underlies differential ability of neuroligins to induce functional inhibitory synapses. J Neurosci 33:3612–3623. 10.1523/JNEUROSCI.1811-12.2013 23426688 PMC6619523

[B10] Futai K, Kim MJ, Hashikawa T, Scheiffele P, Sheng M, Hayashi Y (2007) Retrograde modulation of presynaptic release probability through signaling mediated by PSD-95-neuroligin. Nat Neurosci 10:186–195. 10.1038/nn1837 17237775 PMC4755312

[B11] Grimm D, Streetz KL, Jopling CL, Storm TA, Pandey K, Davis CR, Marion P, Salazar F, Kay MA (2006) Fatality in mice due to oversaturation of cellular microRNA/short hairpin RNA pathways. Nature 441:537–541. 10.1038/nature04791 16724069

[B12] Hoogenraad CC, Popa I, Futai K, Sanchez-Martinez E, Wulf PS, van Vlijmen T, Dortland BR, Oorschot V, Govers R, Monti M, Heck AJ, Sheng M, Klumperman J, Rehmann H, Jaarsma D, Kapitein LC, van der Sluijs P (2010) Neuron specific Rab4 effector GRASP-1 coordinates membrane specialization and maturation of recycling endosomes. PLoS Biol 8:e1000283. 10.1371/journal.pbio.1000283 20098723 PMC2808209

[B13] Jackson AL, Bartz SR, Schelter J, Kobayashi SV, Burchard J, Mao M, Li B, Cavet G, Linsley PS (2003) Expression profiling reveals off-target gene regulation by RNAi. Nat Biotechnol 21:635–637. 10.1038/nbt831 12754523

[B14] Jackson AL, Burchard J, Schelter J, Chau BN, Cleary M, Lim L, Linsley PS (2006a) Widespread siRNA "off-target" transcript silencing mediated by seed region sequence complementarity. RNA 12:1179–1187. 16682560 10.1261/rna.25706 PMC1484447

[B15] Jackson AL, Burchard J, Leake D, Reynolds A, Schelter J, Guo J, Johnson JM, Lim L, Karpilow J, Nichols K, Marshall W, Khvorova A, Linsley PS (2006b) Position-specific chemical modification of siRNAs reduces "off-target" transcript silencing. RNA 12:1197–1205. 16682562 10.1261/rna.30706 PMC1484422

[B16] Kariko K, Bhuyan P, Capodici J, Weissman D (2004) Small interfering RNAs mediate sequence-independent gene suppression and induce immune activation by signaling through toll-like receptor 3. J Immunol 172:6545–6549. 10.4049/jimmunol.172.11.6545 15153468

[B17] Khan AA, Betel D, Miller ML, Sander C, Leslie CS, Marks DS (2009) Transfection of small RNAs globally perturbs gene regulation by endogenous microRNAs. Nat Biotechnol 27:549–555. 10.1038/nbt.1543 19465925 PMC2782465

[B18] Lo DC, McAllister AK, Katz LC (1994) Neuronal transfection in brain slices using particle-mediated gene transfer. Neuron 13:1263–1268. 7993619 10.1016/0896-6273(94)90412-x

[B19] Mah LJ, El-Osta A, Karagiannis TC (2010) gammaH2AX: a sensitive molecular marker of DNA damage and repair. Leukemia 24:679–686. 10.1038/leu.2010.6 20130602

[B20] Mäkinen PI, Koponen JK, Kärkkäinen AM, Malm TM, Pulkkinen KH, Koistinaho J, Turunen MP, Ylä-Herttuala S (2006) Stable RNA interference: comparison of U6 and H1 promoters in endothelial cells and in mouse brain. J Gene Med 8:433–441. 10.1002/jgm.860 16389634

[B21] McAllister AK (2000) Biolistic transfection of neurons. Sci STKE 2000:pl1. 10.1126/stke.2000.51.pl1 11752611

[B22] Pologruto TA, Sabatini BL, Svoboda K (2003) ScanImage: flexible software for operating laser scanning microscopes. Biomed Eng Online 2:13. 10.1186/1475-925X-2-13 12801419 PMC161784

[B23] Sledz CA, Holko M, de Veer MJ, Silverman RH, Williams BR (2003) Activation of the interferon system by short-interfering RNAs. Nat Cell Biol 5:834–839. 10.1038/ncb1038 12942087

[B24] Stoppini L, Buchs PA, Muller D (1991) A simple method for organotypic cultures of nervous tissue. J Neurosci Methods 37:173–182. 1715499 10.1016/0165-0270(91)90128-m

[B25] Wakita Y, Kakimoto T, Katoh H, Negishi M (2011) The F-BAR protein Rapostlin regulates dendritic spine formation in hippocampal neurons. J Biol Chem 286:32672–32683. 10.1074/jbc.M111.236265 21768103 PMC3173151

[B26] Zhang H, Macara IG (2006) The polarity protein PAR-3 and TIAM1 cooperate in dendritic spine morphogenesis. Nat Cell Biol 8:227–237. 10.1038/ncb1368 16474385

